# A whole food, plant-based randomized controlled trial in metastatic breast cancer: feasibility, nutrient, and patient-reported outcomes

**DOI:** 10.1007/s10549-024-07284-z

**Published:** 2024-03-30

**Authors:** Erin K. Campbell, Thomas M. Campbell, Eva Culakova, Lisa Blanchard, Nellie Wixom, Joseph J. Guido, James Fetten, Alissa Huston, Michelle Shayne, Michelle C. Janelsins, Karen M. Mustian, Richard G. Moore, Luke J. Peppone

**Affiliations:** 1 Department of Public Health Sciences, University of Rochester Medical Center, 265 Crittenden Blvd, Rochester, NY 14642 USA; 2 Department of Family Medicine, University of Rochester Medical Center, Rochester, NY USA; 3 Department of Surgery, Cancer Control, University of Rochester Medical Center, Rochester, NY USA; 4 Clinical Research Center, University of Rochester Medical Center, Rochester, NY USA; 5 Memorial Sloan Kettering Cancer Center, Westchester, NY USA; 6 Department of Medicine, Hematology/Oncology, University of Rochester Medical Center, Rochester, NY USA; 7 Department of Obstetrics and Gynecology, University of Rochester Medical Center, Rochester, NY USA

**Keywords:** Metastatic breast cancer, Breast cancer, Obesity, Quality of life, Patient-reported outcomes, Nutrition, Plant-based, Vegan diet

## Abstract

**Purpose:**

Quality of life (QOL) is among the most important outcomes for women with metastatic breast cancer (MBC), and it predicts survival. QOL is negatively impacted by cognitive impairment, fatigue, and weight gain. We assessed whether a whole food, plant-based (WFPB) diet-promoting weight loss is feasible and might improve QOL.

**Methods:**

Women with MBC on stable systemic treatments were randomized 2:1 to 1) WFPB dietary intervention (*n* = 21) or 2) usual care (*n* = 11) for 8 weeks. Participants attended weekly education visits and consumed an ad libitum WFPB diet (3 prepared meals/day provided). Patient-reported outcomes and 3-day food records were assessed at baseline and 8 weeks. The effects of WFPB diet on changes in outcomes were assessed by analysis of covariance model controlling for baseline.

**Results:**

20 intervention and 10 control participants completed the trial. Intervention participants were highly adherent to the WFPB diet (94.3 % total calories on-plan). Intervention group nutrient intakes changed significantly including dietary fat (35.8 % to 20.4 % percent calories from fat, *p* < 0.001) and fiber content (12.7 to 30.8 g fiber/1000 kcal, *p* < 0.001). Perceived cognitive function (FACT-Cog total + 16.1; 95 % confidence interval [CI] = 0.8–31.7; *p* = 0.040) and emotional well-being (FACT-B emotional well-being subscale + 2.3; CI = 0.5–4.1; *p* = 0.016) improved in the WFPB versus the control group. Fatigue, measured by the BFI, improved within the WFPB group for fatigue severity (M = 4.7 ± 2.5[SD] to 3.7 ± 2.3, *p* = 0.047) and fatigue at its worst (5.8 ± 2.8 to 4.4 ± 2.4, *p* = 0.011).

**Conclusions:**

Significant dietary changes in this population are feasible and may improve QOL by improving treatment-related symptoms. Additional study is warranted.

Trial Registration: ClinicalTrials.gov identifier: NCT03045289. Registered 7 February 2017.

## Background

Breast cancer (BC) is the most common cancer other than nonmelanoma skin cancer [1], and increasing numbers of women are living with advanced stage breast cancer [2, 3]. Goals of care include reducing risks of cancer progression and mortality, but also preserving or increasing quality of life (QOL). In fact, QOL is one of the most important outcomes to women with metastatic breast cancer (MBC) [4–6], and recent evidence shows both QOL and patient-reported symptom burden predict survival in MBC patients [7–10].

QOL is strongly affected by treatment-related symptoms [11]: cognitive impairment, fatigue, and weight gain. Self-reported cognitive impairment occurs in 45 % of women receiving systemic therapy [12], and cancer-related fatigue (CRF) adversely impacts QOL by reducing activities of daily living [13]. Obesity, associated with a lower QOL [14], fatigue [15], and persistent cognitive changes [16] and complaints [17], is common among BC patients. The prevalence of obesity in MBC patients mirrors its overall population prevalence, which in U.S. adults is above 42 % [18, 19].

Dietary intervention may affect treatment-related symptoms by affecting body weight and inflammation, which, in turn, are associated with QOL [14, 15]. MBC patients with obesity have been found to have significantly higher levels of inflammatory markers than MBC patients without obesity [14]. Evidence suggests that weight loss [20] and dietary change [21] might reduce systemic inflammation leading to improvements in cognitive function [22, 23] and fatigue [24] and thereby QOL. A whole food, plant-based (WFPB) diet, exclusively comprised of minimally processed plant foods, has been demonstrated to result in significant, clinically meaningful weight loss [25] and significantly lower inflammatory marker levels [26, 27]. While some suggest that weight loss alone can improve QOL [28], the effect of intentional weight loss on cancer-related outcomes in women with MBC remains unexplored [29].

Given the plausible benefits and lack of prior dietary interventions in this population, we designed and conducted a pilot study to explore feasibility and preliminary outcomes. Our intervention resulted in significant improvement of cardiometabolic and hormonal markers and intentional weight loss, described in a separate publication [30]. This paper reports our feasibility results, including dietary adherence, changes in nutritional intake, and patient-reported outcomes (PROs).

## Methods

This study was performed in compliance with recognized ethical guidelines, including the U.S. Common Rule. The study protocol was approved by the University of Rochester Research Subject Review Board (ClinicalTrials.gov identifier: NCT03045289; registered 7 February 2017), and written informed consent was obtained from all participants. Women with MBC were recruited from oncology clinics at the University of Rochester Medical Center and via flyers at local support groups. Women age greater than 18 years with stage 4 BC with any ER/PR/HER2 status expected to live at least 6 months by their oncologist and on a stable treatment regimen for ≥ 6 weeks with no expected near future changes were eligible. Exclusions included inability to tolerate a normal diet, active malabsorption syndrome or eating disorder, uncontrolled diarrhea, recent vegan diet, major surgery within 2 months, current insulin, sulfonylurea, or warfarin use, glomerular filtration rate < 30 mL/min/1.73 m^2^, or serum potassium level > 5.3 mmol/L twice within 90 days, current smoking, illicit drug use, more than 7 alcoholic drinks/week, plant-based food allergies or intolerances, or psychiatric disorder impairing ability to give consent. After consent, additional screening consisted of attendance at an informational session providing an overview of the study, sampling provided study food, and an individual follow-up visit with the study physicians. If a participant consented but did not complete screening procedures or chose not to proceed with participation, she was deemed a screen failure.

At the conclusion of participants’ individual visits, participants were randomized 2:1 to two arms: 1) WFBP diet (*n* = 21) or 2) usual diet control (*n* = 11) by computer algorithm with blocks of size 4. Participants in the WFPB arm received 3 prepared meals and one side dish per day for 8 weeks, weekly education with the study physicians (TMC and EKC), and a weekly phone call from one of the study physicians. The ad libitum WFPB diet consisted of fruits, vegetables, whole grains, legumes, nuts, and seeds. Soy foods were allowed as were minimal amounts of added sugars. The diet excluded all animal products, added oils, and solid fats. Participants were encouraged to eat as often and as much as desired to be full. They were allowed to add their own ‘on-plan’ food in addition to, or in place of, the provided food. The provided meals were not designed to provide a specific calorie amount or nutrient intake, but rather to enhance dietary adherence. A daily multivitamin (Centrum Women) was provided to all participants for the 8 week trial duration.

Education and coaching consisted of one office visit/week for the duration of the 8 week trial, conducted in person (pre-COVID) or via remote teleconferencing (post-COVID) (Zoom Video Communications, Inc., San Jose, CA) with TMC and EKC. In addition to individual assessments and coaching, educational topics included discussions of the food guide, shopping guide, recipes, and label reading, as well as discussing the effects of nutrition on weight loss, cardiovascular health, blood glucose, and behavioral change topics (changing tastes, cravings, and willpower). Between visits, brief weekly telephone calls were made to offer additional coaching and/or helpful resources.

Participants in the usual diet control arm were instructed to continue their usual diets for 8 weeks and received phone calls from a study physician at weeks 2 and 6 to assess for adverse events and treatment changes. As an incentive to maintain participation, control participants received condensed WFPB educational resources and 2 weeks of prepared study meals after completing their final 8-week assessments.

Dietary assessments included two 3 day food records (two weekdays and one weekend day at baseline and week 8) and three unscheduled 24 h recalls. Unscheduled 24 h food recalls were conducted by phone by a dietitian (NW) at approximately 2, 4, and 6 weeks. Three-day food records and 24 h recalls were analyzed using Nutrition Data System for Research (NDSR), version 2017 (Nutrition Coordinating Center, University of Minnesota, Minneapolis, MN). Dietary compliance was calculated as the percentage of calories consumed from on-plan foods. On-plan was predefined as foods and meals that did not contain added liquid oils, solid fats, or animal-based products. By our predefined criteria, a participant was adherent if she consumed at least 80 % of her calories from on-plan foods and attended at least 6 of 8 weekly visits.

Participants completed questionnaires at baseline and 8 weeks. Validated questionnaires included the Brief Fatigue Inventory (BFI), European Organization for the Research and Treatment of Cancer Quality of Life Questionnaire (EORTC QLQ-C30), Functional Assessment of Cancer Therapy—Breast (FACT-B), Functional Assessment of Cancer Therapy—Cognitive Function (FACT-Cog), and a modified M.D. Anderson Cancer Center Symptom Inventory (https://www.mdanderson.org/research/departments-labs-institutes/departments-divisions/symptom-research/symptom-assessment-tools/md-anderson-symptom-inventory.html). Participants completed a demographic questionnaire at baseline and a feedback questionnaire at 8 weeks.

### Statistical analysis

Between arm balance of participants’ clinical and sociodemographic characteristics was evaluated, and relevant descriptive statistics (mean, standard deviation (SD)) were generated. Distribution of outcome variables was assessed at baseline and 8 weeks by study arm graphically and by the descriptive statistics. Within-group change in outcome values from baseline to 8 weeks within each study arm was assessed by paired *t* test. Analysis of covariance models with arm as the main factor and corresponding baseline levels as the covariate was used to evaluate the effects of the WFPB intervention on PROs at 8 weeks and to estimate the mean between-group difference in change in PRO score from baseline. To account for some deviation from the normality assumption, the results were confirmed in non-parametric sensitivity analysis. Statistical significance was set at two-sided alpha = 0.05 level. Data were analyzed using SAS version 9.4 (SAS Inc, Cary, NC, USA).

## Results

Table 1 displays baseline characteristics of participants. The mean BMI of participants was 29.6 kg/m^2^ with 71 % of participants in either the overweight or obese BMI range. The majority of participants had hormone receptor positive BC. Metastatic disease to the bone was the most common site of metastasis, present in 84 % of participants. The most common treatment regimens were a cyclin-dependent kinase 4/6 inhibitor combined with an aromatase inhibitor.

**Table 1:** Baseline characteristics

Characteristics	Mean ± SD	Control (*n* = 10 *)	Intervention (n = 21)
Age (years)		64.2 ± 8.9	59.1 ± 11.0
Race	Black, *n* (%)	0 (0)	1 (4.8)
	White, *n* (%)	10 (100.0)	19 (90.5)
	No answer, *n* (%)	0 (0)	1 (4.8)
Ethnicity	Not Hispanic/Latino, *n* (%)	10 (100.0)	20 (95.2)
	No answer, *n* (%)	0 (0)	1 (4.8)
Marital status	Married, *n* (%)	7 (70.0)	14 (66.7)
	Divorced, *n* (%)	2 (20.0)	3 (14.3)
	Single, *n* (%)	1 (10.0)	3 (14.3)
	Widowed, *n* (%)	0 (0)	1 (4.8)
Employment status	Currently employed outside home, *n* (%)	3 (30.0)	6 (28.6)
	Self-employed, *n* (%)	0 (0)	2 (9.5)
	Retired, *n* (%)	4 (40.0)	4 (19.0)
	Disability, *n* (%)	1 (10.0)	3 (14.3)
	Homemaker, *n* (%)	2 (20.0)	4 (19.0)
	Not Working—Other, *n* (%)	0 (0)	2 (9.5)
BMI at study baseline (kg/m^2^)	Mean ± SD	28.4 ± 4.4	30.2 ± 7.2
Age at first breast cancer diagnosis (years)	Mean ± SD	52.9 ± 11.7	49.4 ± 10.9
Years elapsed since first diagnosis	Mean ± SD	11.2 ± 7.9	9.7 ± 6.4
Years elapsed since diagnosis of metastatic breast cancer	Mean ± SD	5.3 ± 6.0	2.2 ± 1.8
Hormone receptor status	ER +, *n* (%)	10 (100.0)	20 (95.2)
	PR +, *n* (%)	9 (90.0)	17 (81.0)
	HER2 +, *n* (%)	3 (30.0)	6 (28.6)
Location of metastases	Bone, *n* (%)	7 (70.0)	19 (90.5)
	Lung, *n* (%)	4 (40.0)	8 (38.1)
	Brain, *n* (%)	1 (10.0)	3 (14.3)
	Liver, *n* (%)	2 (20.0)	1 (4.8)
	Other, *n* (%)	6 (60.0)	7 (33.3)
Cancer therapy	Palbociclib, *n* (%)	3 (30.0)	10 (47.6)
	Abemaciclib, *n* (%)	1 (10.0)	2 (9.5)
	Ribociclib, *n* (%)	0 (0)	1 (4.8)
	Trastuzumab, *n* (%)	2 (20.0)	5 (23.8)
	Pertuzumab, *n* (%)	1 (10.0)	4 (19.0)
	Capecitabine, *n* (%)	1 (10.0)	1 (4.8)
	Letrozole, *n* (%)	3 (30.0)	13 (61.9)
	Anastrozole, *n* (%)	3 (30.0)	1 (4.8)
	Fulvestrant, *n* (%)	2 (20.0)	3 (14.3)
	Exemestane, *n* (%)	1 (10.0)	2 (9.5)
	Denosumab, *n* (%)	1 (10.0)	10 (47.6)
	Zoledronic acid, *n* (%)	0 (0)	1 (4.8)
	Leuprolide, *n* (%)	0 (0)	2 (9.5)

* One control subject was lost to follow up immediately after randomization to the control group

Her baseline characteristics were incomplete and not reported here

*SD* standard deviation, *BMI* body mass index

### Feasibility

**Fig. 1:**
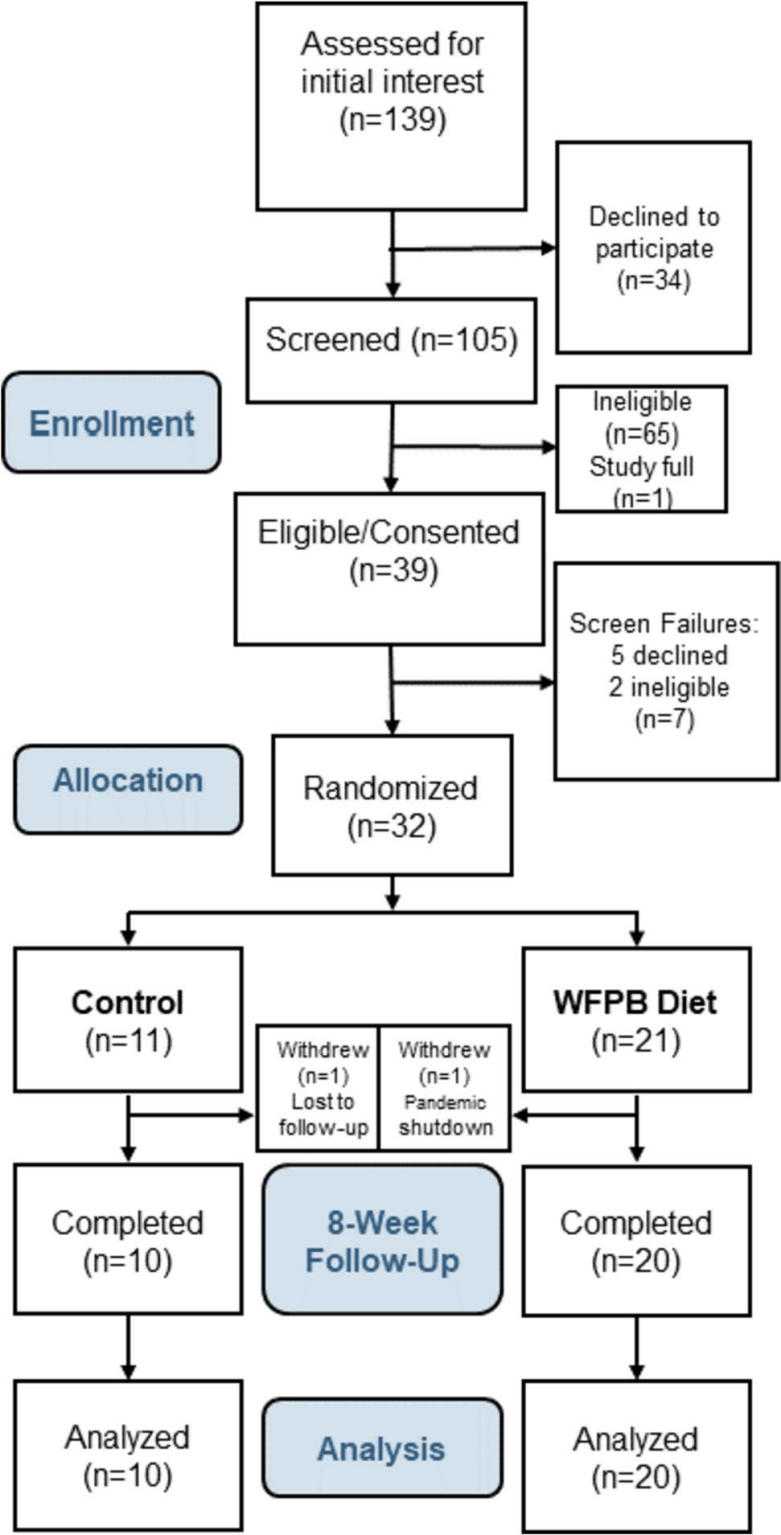
Consort diagram

Thirty of 32 randomized participants completed their study participation. One participant was lost to follow-up immediately following randomization to the control arm. One intervention participant was withdrawn by investigators in March 2020 shortly after baseline, due to the COVID-19 pandemic shutdown (Fig. 1). All 30 remaining participants completed assessments at 8 weeks. All (100 %) intervention participants attended at least 6 of the 8 weekly visits, our prespecified criterion for study visit adherence.

Intervention participants were highly adherent to the diet. Eighteen of 19 (94.7 %) intervention participants with complete dietary assessments met the study’s prespecified adherence criterion that they derive ≥ 80 % of their kilocalories from on-plan foods. In fact, 94.3 % of total calories consumed by the intervention participants with complete dietary assessments were from on-plan foods (foods without added oils, solid fats, or animal-based ingredients). Changes in dietary intake between baseline and final 3-day food records are shown in Table 2.

**Table 2:** Nutrient intake of intervention and control groups at baseline and 8 weeks

	Intervention group (*n* = 19)***		Control group (*n* = 10)		Between group (adjusted for baseline)	
	Baseline	Week 8 Final	Baseline	Week 8 Final	Difference in change	*p*- value
Energy (kcal)	1782.3 ± 330.4	1321.3 ± 262.4 **	1590.2 ± 442.7	1431.2 ± 401.0 *	− 301.9 ± 285.7	< 0.001
Fat (% of total kcal)	35.8 ± 6.2	20.4 ± 5.8 **	35.0 ± 5.8	34.8 ± 11.9	− 15.2 ± 8.9	0.012
Carbohydrate (% of total kcal)	48.4 ± 7.3	66.2 ± 8.0 **	44.8 ± 10.0	45.4 ± 17.7	17.3 ± 11.4	< 0.001
Protein (% of total kcal)	14.6 ± 2.5	12.6 ± 2.9 *	19.3 ± 9.7	18.8 ± 7.8	− 1.5 ± 4.6	0.490
% total protein provided by plant sources	46.6 ± 11.3	95.7 ± 11.8 **	45.5 ± 25.9	43.0 ± 21.9	51.6 ± 14.7	< 0.001
Dietary Cholesterol (mg)	214.2 ± 104.6	7.5 ± 18.3 **	217.1 ± 139.5	188.2 ± 119.6	− 177.8 ± 133.3	0.002
Dietary Fiber (g/1000 kcal)	12.7 ± 5.7	30.8 ± 5.6 **	16.5 ± 6.0	14.9 ± 3.9	19.7 ± 6.4	< 0.001

*** One intervention participant was missing the final 3 day food record

*p < 0.05 for within-group change

**p < 0.01 for within-group change

Within the intervention group, dietary intake changed significantly. Intervention participants consumed 25.9 % fewer calories (*p* < 0.001). Fat as a percentage of total kilocalories and dietary cholesterol intake were significantly reduced by 43.0 % (*p* < 0.001) and 96.5 % (*p* < 0.001), respectively. Carbohydrates as a percentage of total kilocalories increased 36.8 % (*p* < 0.001) and dietary fiber per 1000 kcal increased 242.5 % (*p* < 0.001). Protein as a percentage of total kilocalories was modestly reduced (− 13.7 %, *p* < 0.017). The proportion of total protein provided by plant sources increased 105.4 % (*p* < 0.001).

The composition of the control group’s diet was largely unchanged despite knowing that we were studying a WFPB diet. Within the control group, participants reduced their total calories (− 10.0 %, *p* < 0.034). There were no statistically significant changes to the percent of total calories from any of the macronutrients, dietary cholesterol, the proportion of protein provided by plant sources, or dietary fiber per 1000 kcal.

The groups differed significantly in their mean nutrient changes from baseline to week 8 in all nutrients displayed in Table 2 except for change in percent of calories from protein.

Of note, there was a significant between-group effect (*p* < 0.01); intervention group participants lost a mean of 6.6 % of their body weight, whereas control group participants lost a mean of 0.7 % of their body weight. When adjusted for baseline weight, intervention participants lost 9.0 pounds more than control participants (*p* < 0.001). Changes in BMI, cardiometabolic outcomes, and hormonal markers are described in a separate publication [30].

### Adverse events

Adverse events during the trial were infrequent and mild. Grade 2 hypotension occurred in three intervention participants. In each case, symptoms were mild and resolved after referral to their routine medical providers for medication adjustments. One control participant reported lightheadedness following a blood draw. Other adverse events included aphthous ulcer, transient, mild hyponatremia, and mild, transient neutropenia, all deemed medication related. One participant in each arm had her primary cancer treatment dose reduced by her oncologist due to adverse events typical of that medication.

### Patient-reported outcomes

Table 3 shows the effect of the intervention arms on various patient-reported outcomes. The total FACT-B total score (+8.0, *p* < 0.001), as well as physical (+1.5, p = 0.016) and emotional well-being (+1.8, p = 0.012), and BC-specific symptoms (+3.2, p = 0.002) subscale scores improved significantly within the intervention group; surpassing the clinically important difference (CID) for each of these measures established in prior studies [31–33]. The change in emotional well-being score from baseline to 8 weeks was significantly different between the groups (+2.3, CID = 0.9 [31]; p = 0.016, ES = 0.54). The between-group difference in the FACT-B score approached significance (+5.1, CID = 6.0 [32, 33]; p = 0.067, ES = 0.29).

**Table 3:** Patient-reported outcomes

			Intervention Group (*n* = 20)		Control Group (*n* = 10)		Between-Group Difference Mean ± SE	Between Group Effect Size^a^	*p*-value (between-group)^b^
Questionnaire		Better Score	Baseline Mean ± SD	Final Mean ± SD	Baseline Mean ± SD	Final Mean ± SD			
**FACT-Cog total**			**140.8 ± 32.0**	156.6 ± 29.6 **	**146.4 ± 40.6**	**145.1 ± 41.8**	**16.1 ± 7.4**	**0.46**	**0.040**
	Perceived cognitive impairment	Higher	81.3 ± 21.6	90.8 ± 19.0 **	84.3 ± 24.2	84.7 ± 24.8	8.4 ± 4.6	0.37	0.076
	Perceived cognitive abilities	Higher	24.9 ± 9.6	27.7 ± 6.7	28.7 ± 7.9	29.1 ± 7.9	0.7 ± 1.9	0.08	0.701
	**Comments from others**	**Higher**	**10.2 ± 2.1**	11.2 ± 1.3 *	**10.5 ± 2.1**	**9.8 ± 3.0**	**1.6 ± 0.6**	**0.79**	**0.006**
	**Impact of perceived cognitive impairments on QOL**	**Higher**	**22.0 ± 7.4**	25.1 ± 6.5 *	**22.9 ± 9.0**	**21.5 ± 8.6**	**4.1 ± 2.0**	**0.52**	**0.049**
Symptom Inventory									
	**Problems remembering things**	**Lower**	**3.2 ± 2.6**	2.0 ± 1.4 **	**1.6 ± 2.0**	**2.6 ± 3.3**	**− 1.6 ± 0.7**	**− 0.67**	**0.024**
	**Problems concentrating**	**Lower**	**2.75 ± 2.5**	1.9 ± 1.6 *	**1.8 ± 2.7**	**2.6 ± 3.1**	**− 1.3 ± 0.6**	**− 0.50**	**0.039**
	Problems paying attention	Lower	2.3 ± 2.3	1.5 ± 1.8	1.8 ± 2.7	2.5 ± 3.2	− 1.3 ± 0.7	− 0.54	0.056
	**Problems multitasking**	**Lower**	**2.0 ± 2.2**	**1.6 ± 1.7**	**1.9 ± 2.0**	**3.1 ± 3.5**	**− 1.5 ± 0.7**	**− 0.73**	**0.029**
	Fatigue	Lower	4.6 ± 2.1	3.6 ± 2.4	3.8 ± 3.1	3.9 ± 2.4	− 0.8 ± 0.8	− 0.31	0.334
	Shortness of breath	Lower	1.8 ± 2.4	1.1 ± 2.3 *	1.5 ± 2.4	1.2 ± 1.8	− 0.3 ± 0.5	− 0.12	0.560
	**Diarrhea**	**Lower**	**1.5 ± 2.8**	**0.75 ± 1.4**	**0.5 ± 1.6**	**2.2 ± 3.4**	**− 1.8 ± 0.8**	**− 0.72**	**0.044**
	**Skin problems**	**Lower**	**0.8 ± 2.1**	**0.5 ± 1.7**	**1.0 ± 2.5**	**1.3 ± 2.3**	**− 0.6 ± 0.2**	**− 0.28**	**0.007**
	Symptoms interfered with general physical activity	Lower	2.6 ± 2.6	1.8 ± 2.2	2.6 ± 2.3	2.5 ± 3.0	− 0.7 ± 0.6	− 0.29	0.259
	Symptoms interfered with walking	Lower	2.1 ± 2.5	1.2 ± 2.1 *	1.9 ± 2.9	1.3 ± 2.4	− 0.2 ± 0.5	− 0.09	0.627
	Symptoms interfered with QOL	Lower	2.7 ± 2.6	1.7 ± 2.2	2.0 ± 2.4	2.3 ± 2.7	− 1.1 ± 0.7	− 0.41	0.126
FACT-B total		Higher	103.1 ± 14.8	111.0 ± 14.0 *	109.7 ± 21.8	111.2 ± 17.1	5.1 ± 2.7	0.29	0.067
	Physical well-being	Higher	21.4 ± 4.3	22.8 ± 4.5 *	22.6 ± 5.8	22.1 ± 5.1	1.7 ± 1.0	0.36	0.093
	Social/family well-being	Higher	21.8 ± 4.8	22.6 ± 4.5	22.9 ± 4.3	24.1 ± 4.3	− 0.6 ± 1.2	− 0.14	0.611
	**Emotional well-being**	**Higher**	**16.2 ± 4.2**	18.0 ± 3.3 *	**18.1 ± 4.1**	**16.8 ± 3.2**	**2.3 ± 0.9**	**0.54**	**0.016**
	Functional well-being	Higher	18.0 ± 5.2	18.7 ± 5.0	19.1 ± 7.0	20.7 ± 5.3	− 1.2 ± 1.1	− 0.20	0.283
	Breast cancer subscale	Higher	25.8 ± 4.0	28.9 ± 3.7 **	27.0 ± 5.0	27.5 ± 4.5	2.1 ± 1.3	0.49	0.102
BFI global fatigue		Lower	3.4 ± 2.2	2.6 ± 2.1	2.6 ± 2.8	2.3 ± 2.0	− 0.2 ± 0.6	− 0.07	0.779
	Fatigue severity	Lower	4.7 ± 2.5	3.7 ± 2.3 *	3.5 ± 3.1	3.2 ± 1.6	0.01 ± 0.7	0.01	0.984
	Fatigue at its worst	Lower	5.8 ± 2.8	4.4 ± 2.4 *	4.3 ± 3.4	3.7 ± 2.0	− 0.01 ± 0.7	− 0.004	0.986
EORTC QLQ-C30									
	Global health status/QOL	Higher	67.9 ± 22.0	71.3 ± 23.5	75 ± 16.1	79.6 ± 11.9	− 3.1 ± 5.8	− 0.15	0.600
	Physical functioning	Higher	77.2 ± 20.8	78.3 ± 22.8	78.5 ± 27.0	73.3 ± 24.5 *	6.1 ± 5.0	0.27	0.234
	Role functioning	Higher	80.0 ± 27.9	81.7 ± 24.7	68.3 ± 30.9	75.0 ± 30.7	− 2.1 ± 6.4	− 0.07	0.744
	Emotional functioning	Higher	67.9 ± 19.4	74.2 ± 20.0	74.2 ± 28.7	79.2 ± 19.7	− 1.8 ± 6.4	− 0.08	0.786
	Cognitive functioning	Higher	73.3 ± 19.8	84.2 ± 15.7 **	76.7 ± 31.6	75.0 ± 29.7	11.3 ± 5.8	0.47	0.061
	Social functioning	Higher	71.1 ± 24.1	80.7 ± 21.7 *	75.0 ± 25.2	83.3 ± 22.2	− 0.14 ± 6.2	− 0.01	0.983
	Fatigue	Lower	43.9 ± 23.5	34.4 ± 22.8 *	37.8 ± 28.3	35.6 ± 26.1	− 5.6 ± 6.1	− 0.22	0.370
	Insomnia	Lower	35.0 ± 27.5	21.7 ± 27.1	20.0 ± 32.2	26.7 ± 34.2	− 11.7 ± 10.8	− 0.40	0.289

*p < 0.05 for within-group change

**p < 0.01 for within-group change

Bold rowsare questionnaires or subsets of questionnaires with p<0.05 for between-group differences

^a^Effect size was calculated as difference relative to baseline standard deviation

^b^*p* value is assessing the between-group difference in change from the baseline to 8 weeks in analysis of covariance model

*SD* standard deviation, *SE* standard error, *FACT-Cog* Functional Assessment of Cancer Therapy—Cognitive Function, *Symptom Inventory* M.D. Anderson Cancer Center Symptom Inventory, *FACT-B* Functional Assessment of Cancer Therapy—Breast, *BFI* Brief Fatigue Inventory, *EORTC QLQ-C30* European Organization for the Research and Treatment of Cancer Quality of Life Questionnaire

Perceived cognitive function, as measured by the FACT-Cog questionnaire, showed clinically significant improvements within the intervention group as well as between the groups. (Table 3). Between groups, there were statistically and clinically significant improvements on the overall FACT-Cog score (+ 16.1, CID = 9.6 [34, 35]; *p* = 0.04, ES = 0.46) as well as on the comments from others subscale (+ 1.6, CID = 0.4 [35]; *p* = 0.006, ES = 0.79) and the impact of perceived cognitive impairments on QOL subscale (+ 4.1, CID = 0.9 [35]; *p* = 0.049, ES = 0.52) in the intervention group compared to the control. Perceived cognitive impairment improved in the intervention group (*p* = 0.007) and the between-group difference in change from the baseline to 8 weeks approached significance (+ 8.4, CID = 5.5 [34]; *p* = 0.076, ES = 0.37). Improvement in the perceived cognitive ability subscale approached significance within the intervention group (*p* = 0.056).

On the symptom inventory, there were statistically significant between group differences favoring the intervention group in improvements in problems remembering things (p = 0.024, ES =  − 0.67), problems concentrating (p = 0.039, ES =  − 0.5), and problems multitasking (*p* = 0.029, ES =  − 0.73). Reported problems paying attention trended towards improvement in the intervention group compared to the control group in which the score worsened (*p* = 0.056, ES =  − 0.54). Diarrhea (*p* = 0.044) and skin problems (*p* = 0.007) improved in the intervention group compared to the control. Shortness of breath (*p* = 0.039) and interference of symptoms with physical activity (*p* = 0.057), walking (*p* = 0.020), and QOL (*p* = 0.053) improved within the intervention group. Fatigue trended towards improvement within the intervention group (*p* = 0.054).

Mirroring results of the FACT-Cog questionnaire, there was a significant improvement in perceived cognitive functioning in the intervention group (*p* = 0.004) as assessed by the EORTC QLQ-C30. This difference approached significance between the groups (*p* = 0.061, ES = 0.47). Social functioning (*p* = 0.023) and fatigue (*p* = 0.013) improved significantly in the intervention group, but not between groups. There was a trend towards improved insomnia in the intervention group (*p* = 0.072).

Fatigue, as measured by the BFI, improved in the intervention group. There were significant within-group improvements in fatigue severity (*p* = 0.047) and fatigue at its worst (*p* = 0.011) and a trend towards improvement in global fatigue (*p* = 0.058) for the WFPB group.

### Acceptability

Intervention participants had positive perceptions of the intervention and provided food. When asked “Do you feel that your health has benefited from the study intervention?”, 19 of 20 intervention participants answered “Yes” (95.0 %, 1 did not give an answer). As shown in Table 4, participants strongly recommended the intervention, reported minimal hunger, and positively reviewed both the amount and taste of provided meals.

**Table 4:** Participant perception of intervention (*n* = 20)

	Mean ± SD
“On a scale from 1 to 10, how strongly would you recommend that other cancer patients be given this type of nutrition and support intervention if they were able and willing to participate?” (1 = “Would not recommend; 10 = “Highly recommend”)	9.5 ± 1.2
“On a scale of 1 to 10, please rate the taste of the provided food” (1 = “It was hardly edible”; 10 = “Exceptional taste”)	7.8 ± 1.6
“On a scale of 1 to 10, please rate your overall hunger during the course of this study” (1= “Way too hungry most of the time”; 10= “Satisfied and full most of the time”)	8.6 ± 2.2
“On the following scale (of 1 to 5), please rate the amount of provided food” (1 = “It was never, enough”; 3 = “Just right”; 5 = “Far too much”)	4.1 ± 0.7

## Discussion

The findings of this RCT demonstrate that our WFPB dietary intervention in women being treated for MBC is acceptable and feasible, resulting in significant, large changes in nutrient intakes. Clinically and statistically significant improvements were noted for QOL and treatment-related symptoms including perceived cognitive function, physical and emotional well-being, and fatigue. To our knowledge, this is one of the first dietary intervention RCTs that improved QOL and treatment-related symptoms in women currently receiving anti-neoplastic therapy, as the majority of studies have focused on cancer survivors who completed primary therapy.

Feedback from women in the study was overwhelmingly positive: participants highly recommended the intervention, felt that it had improved their health, and based on participant ratings, taste and hunger were not adherence barriers. Intervention group adherence was excellent for both WFPB diet and weekly visit attendance. The intervention was safe and well tolerated.

The changes in nutrient intake achieved in this intervention are large compared to other dietary interventions in BC survivors. Our study achieved a 25.9 % decrease in energy intake, a 43.0 % decrease in energy from fat, and an 84.6 % increase in dietary fiber grams per day. In the Women’s Healthy Eating and Living (WHEL) randomized trial, the largest dietary changes achieved were a 5.8 % decrease in energy (kilocalories) intake, a 25.6 % decrease in energy from fat, and a 46.4 % increase in dietary fiber from baseline [36]. The Women’s Intervention Nutrition Study (WINS) demonstrated a 13.5 % decrease in energy intake, a 31.4 % decrease in energy from fat, and a 6 % increase in dietary fiber at 12 months [37]. We hypothesize that providing food enhanced dietary adherence and facilitated large nutrient intake changes.

PROs improved across multiple instruments and outcome types, with consistent improvements in overall QOL, physical and emotional well-being, cognitive function, and fatigue. The change in the total FACT-B score within the intervention group surpassed the CID [32, 33], and the between-group difference approached significance. The FACT-B emotional well-being subscale score improved significantly between the groups, surpassing the CID [31]. Between groups, perceived cognitive function improved across three different instruments (FACT-Cog, Symptom Inventory, and EORTC QLQ-C30). Improvements on the total FACT-Cog score surpassed the CID [34, 35]. Improvements in fatigue in the intervention group reached or approached significance on the Symptom Inventory, BFI, and EORTC QLQ-C30.

The large changes to nutrient intakes and subsequent intentional weight loss that occurred over the course of our trial may have positively affected PRO measures and thereby QOL. Participants in the intervention group had lost 6.6 % of their body weight at 8 weeks. Evidence suggests that excess weight negatively affects QOL and that weight loss can improve QOL and cognitive function. Obesity is associated with a lower QOL in both clinically stable ER + MBC patients [14] and general adult populations [38]. In patients undergoing weight loss interventions with obesity but without cancer, improved QOL has been demonstrated status post bariatric surgery but more variably after non-surgical weight loss, possibly due to degree of weight loss [38]. Cognitive function also appears to improve with weight loss in overweight and obese patients without cancer [23]. While weight loss appears to be an important factor, dietary composition itself likely plays a role: despite no significant change in BMI, a pilot study of an isocaloric fatigue reduction diet, rich in produce, whole grains, and omega-3 fatty acid rich foods resulted in a 44 % reduction in fatigue [39]. It is possible that our WFPB dietary intervention improved QOL by facilitating clinically meaningful weight loss in the context of significant changes to dietary composition.

Limitations of this study include its small size, particularly the smaller control group, short duration, and lack of a time and attention control. Given the study limitations, it is not possible to elucidate the exact mechanisms or specific intervention components that produced change, but rather to demonstrate initial feasibility and preliminary results. Strengths include the intensity of the intervention and multiple dietary assessments. Provision of meals and intensive education and follow-up likely enhanced adherence, resulting in large changes in dietary intake.

## Conclusion

This is one of the first dietary RCTs to improve disease-specific QOL and treatment-related symptoms in participants receiving treatment for metastatic disease. Our WFPB dietary intervention is both feasible and acceptable; it resulted in large changes in nutrient intake and clinically significant improvements in QOL and symptoms. Given the growing population of patients with MBC and the negative impact of both cancer and ongoing treatment on QOL, these findings are promising. Further study with trials of longer duration and follow-up is warranted to determine sustainability and durability of these benefits.

## Data Availability

The data underlying this article are available by request at https://gitlab-public.circ.rochester.edu/WFPB-breast-cancer/biomarkers. Email Thomas_campbell@urmc.rochester.edu for access to data and/or materials.

## Abbreviations

QOL: Quality of life
MBC: Metastatic breast cancer
WFPB: Whole food, plant-based
CI: 95 % Confidence interval
BC: Breast cancer
CRF: Cancer-related fatigue
PRO: Patient-reported outcome
BMI: Body mass index
SD: Standard deviation
SE: Standard error
FACT-Cog: Functional Assessment of Cancer Therapy-Cognitive Function
Symptom Inventory: M.D. Anderson Cancer Center Symptom Inventory
FACT-B: Functional Assessment Cancer Therapy-Breast
BFI: Brief Fatigue Inventory
EORTC QLQ- C30: European Organization for the Research and Treatment of Cancer Quality of Life Questionnaire
RCT: Randomized controlled trial
